# Supplementary figures and images for: Accurate classification of major brain cell types using in vivo imaging and neural network processing
Source: PLoS Biol. 2023 Nov 9;21(11):e3002357. doi: 10.1371/journal.pbio.3002357 (PMC10689024; doi:10.1371/journal.pbio.3002357)

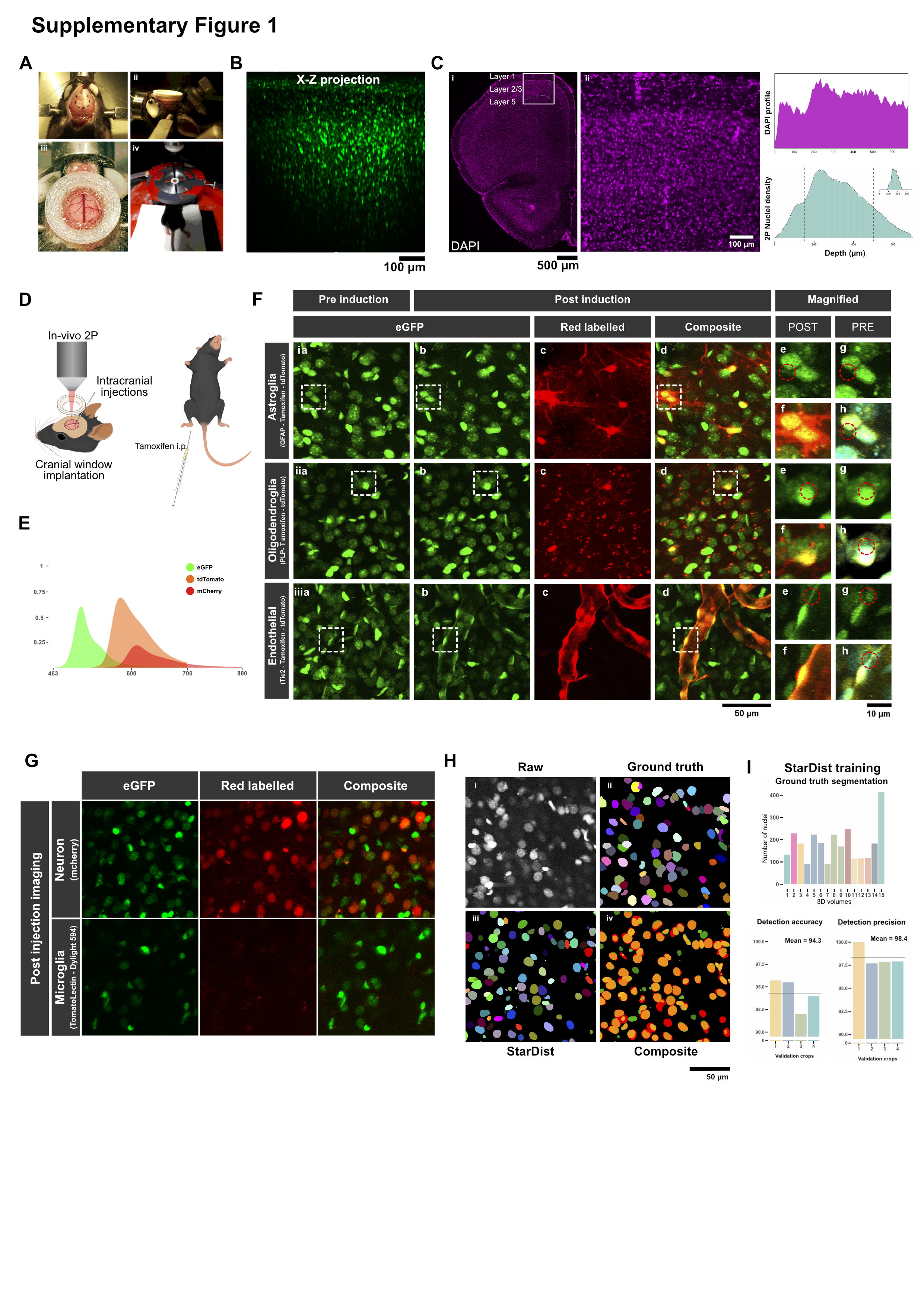

Supplement: S1 Fig — (A) (i–iii) Chronic cranial window implantation using a curved glass cover slip and a custom 3D printed holder. (iv) Fixation of the anesthetized mouse in the custom holder for imaging under the two-photon microscope. (B) X-Z maximum intensity projection of a two-photon volumetric stack (700 μm × 700 μm × 700 μm). (C) Imaging position on a DAPI-stained brain slice. Slice thickness 50 μm. DAPI intensity profile showing similar distribution of intensity as a two-photon image of nuclei. The decreasing nucleus density in the two-photon image stack is a result of attenuation of fluorescence signal at higher depths. Inlay: Nucleus density distribution of sub-volumes used for data analysis. (D) Scheme showing intracranial and intraperitoneal injection strategies for labeling cell types. (E) Fluorescence emission spectra for eGFP, tdTomato, and mCherry (y axis: fluorophore emission normalized to quantum yield, source: fpbase.org). (F) Labeling strategies for red fluorescence expression in reporter mouse lines. Visualization of crosstalk between the eGFP and tdTomato signal after induction with tamoxifen. Overlay of pre (cyan) and post (yellow) GFP after image alignment with the ImageJ plugin bUnwarpJ [33]. (G) No crosstalk is visible between GFP and mCherry signals (upper panel) or eGFP and Tomato lectin-Dylight 594 signals (lower panel). (H) (i) Raw data of H2B-eGFP signal, (ii) manually labeled ground truth, (iii) StarDist segmentation, (iv) composite image of ground truth (red), and StarDist segmentation (green). (I) Upper panel: Count of manually segmented ground truth nuclei for StarDist training, each color depicts an individual mouse. Lower panel: StarDist nucleus detection accuracy and precision, each bar represents an individual imaging volume. Plot data can be found in S1 Data. (TIFF) [file pbio.3002357.s001.tiff]

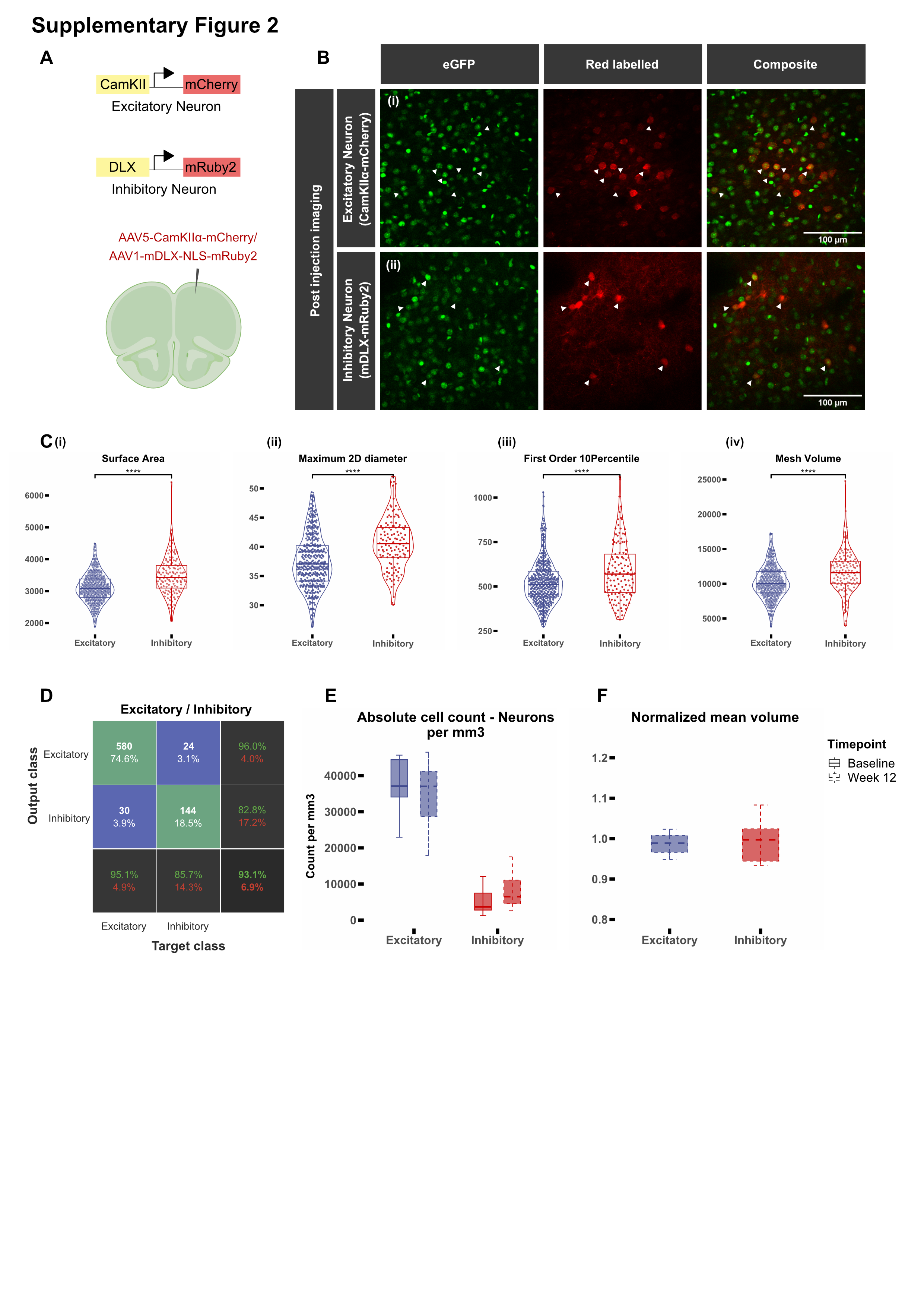

Supplement: S2 Fig — (A) Labeling strategies for excitatory and inhibitory neurons. Intracortical injections were performed at approximately 400 μm deep from the cortex of H2B-eGFP mice using AAV5-CamKIIα-mCherry and AAV1-mDLX-NLS-mRuby2 to visualize excitatory and inhibitory neurons. (B) Post-injection images. Inhibitory neurons were imaged 2–3 weeks after injection; excitatory neurons were imaged 3–4 weeks after injection. (C) Radiomics features showing differences between excitatory and inhibitory neurons for (i) surface area (in pixel 2 [resolution x = 0.29 μm, y = 0.29 μm, z = 2 μm]), (ii) maximum 2D diameter (in pixel, same resolution as in (i)), (iii) first order 10th percentile, (iv) mesh volume in voxels (resolution as in (i)), (n of excitatory neurons: 396, n = 3 mice, n of inhibitory neurons = 122, n = 2 mice). (D) Confusion plot for the classifier. Rows show the predicted class (output class), and the columns show the true class (target class). Green fields illustrate correct identification whereas blue fields illustrate erroneous identifications. The number of observations and the percentage of observations compared to the total number of observations are shown in each cell. Column on the far right shows the precision (or positive predictive value) and false discovery rate in green and red, respectively. Bottom row denotes recall (or true positive rate) and false negative rate in green and red. Cell on the bottom right shows overall accuracy of the classifier. (E) Number of nuclei per mm3 in the secondary motor cortex at baseline after 12 weeks (dashed line) (n = 8 mice). (F) Mean nucleus volume after 12 weeks normalized to baseline (n = 8 mice). (Significance testing for C, E, F: Wilcoxon test, p-values were corrected for multiple comparisons using the Bonferroni method, p < 0.05*, p < 0.01 **, p < 0.001 ***) Plot data can be found in S1 Data. (TIFF) [file pbio.3002357.s002.tiff]

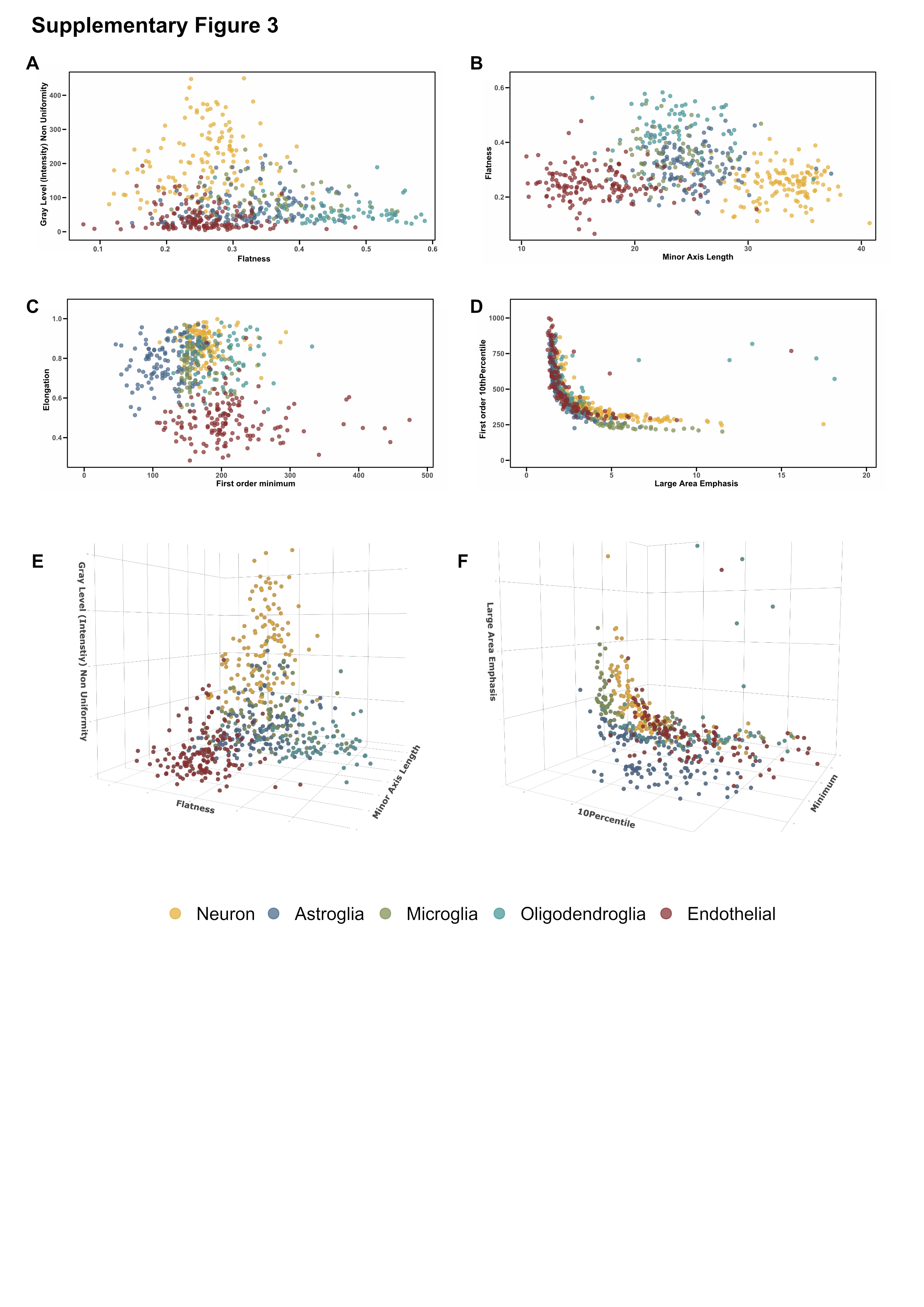

Supplement: S3 Fig — (A) Flatness, gray level non-uniformity. (B) Minor axis length, flatness. (C) First order minimum, elongation. (D) Large area emphasis, first order 10th percentile. (E, F) Combinations of features in 3 dimensions. Plot data can be found in S1 Data. (TIFF) [file pbio.3002357.s003.tiff]

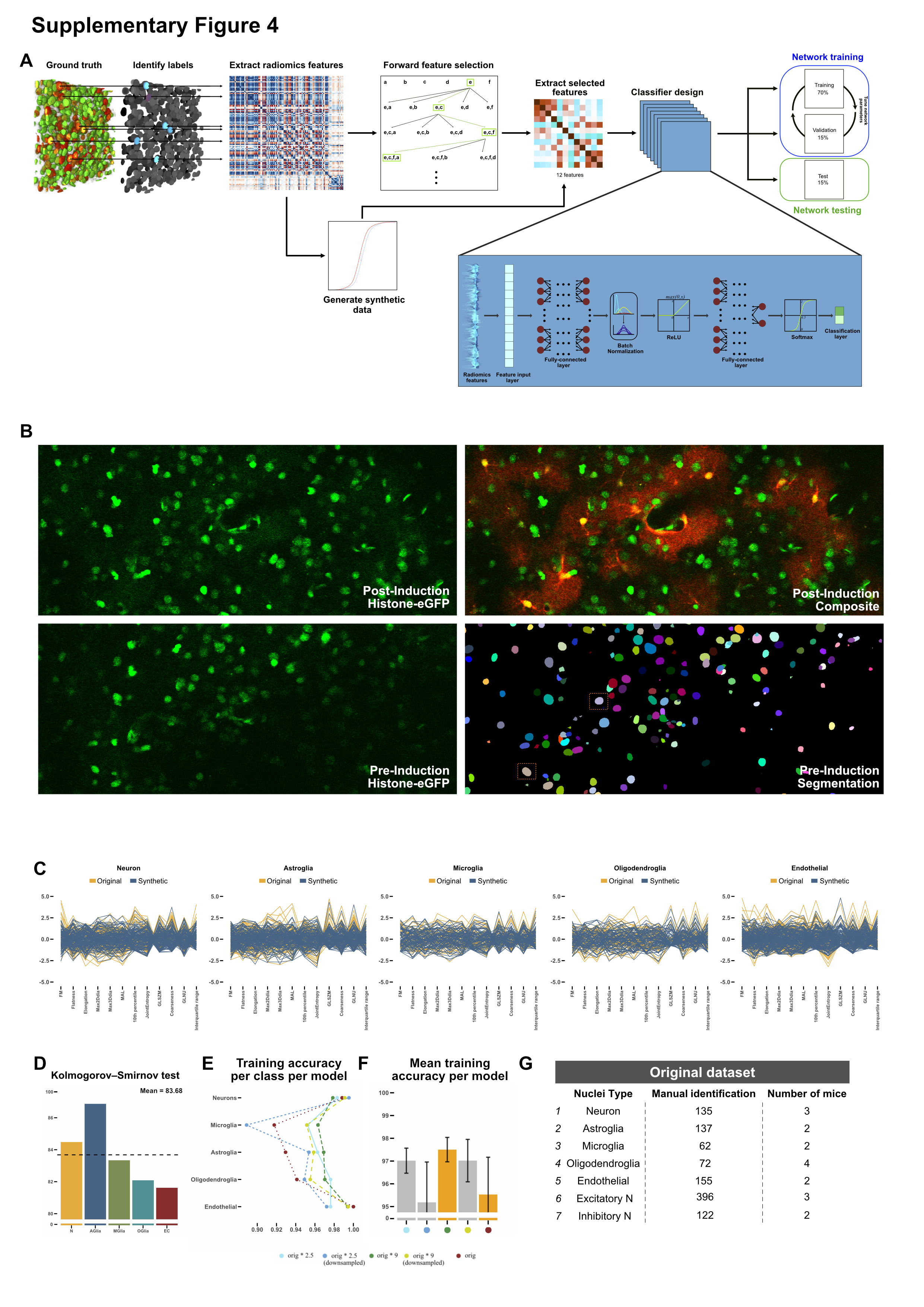

Supplement: S4 Fig — (A) Visualization of the entire classification training process. After ground truth data were selected, a sequential forward feature selection algorithm was applied to extracted features from all nuclei of all cell types, which selected 12 features from the 107 radiomics features. Synthetic data was generated from all the radiomics features of all nuclei and all cell types. The combined dataset was used to train a classifier with the training, validation, and test data comprising 70%, 15%, and 15% of the combined dataset. (B) Example showing manual selection of ground truth data for supervised training. Post-induction green and red channels were overlayed to create a composite. Any nuclei appearing yellow (possessing green and red fluorescence) were identified in the preinduction GFP channel and from its corresponding segmentation, a label id was acquired and later used to identify the extracted features. (C) Synthetic training data generated from the original dataset matched the features of the original datasets. (D) Statistical analysis of similarity between the distribution of original data and distribution of synthetic data (K-S test; mean = 83.68%). (E) Datasets with different amounts of synthetic data were created and training accuracy was compared between them, orig = original dataset, orig * 2.5 = dataset containing 2.5 times the amount of data as the original dataset, orig * 2.5 (down sampled) = dataset down sampled to minimum sample count (after 2.5-fold increase) to equalize sample numbers for all cell types, orig * 9 = dataset containing 9 times the amount of data as the original dataset, orig * 9 (down sampled) = dataset down sampled to minimum sample count (after 9-fold increase) to equalize sample numbers for all cell types. (F) Mean accuracy of classifiers trained 5 times with different combinations of synthetic data; error bars denote standard deviation (SD). Light blue = orig * 2.5, dark blue = orig * 2.5 (downsampled), green = orig * 9, yellow = o [file pbio.3002357.s004.tiff]

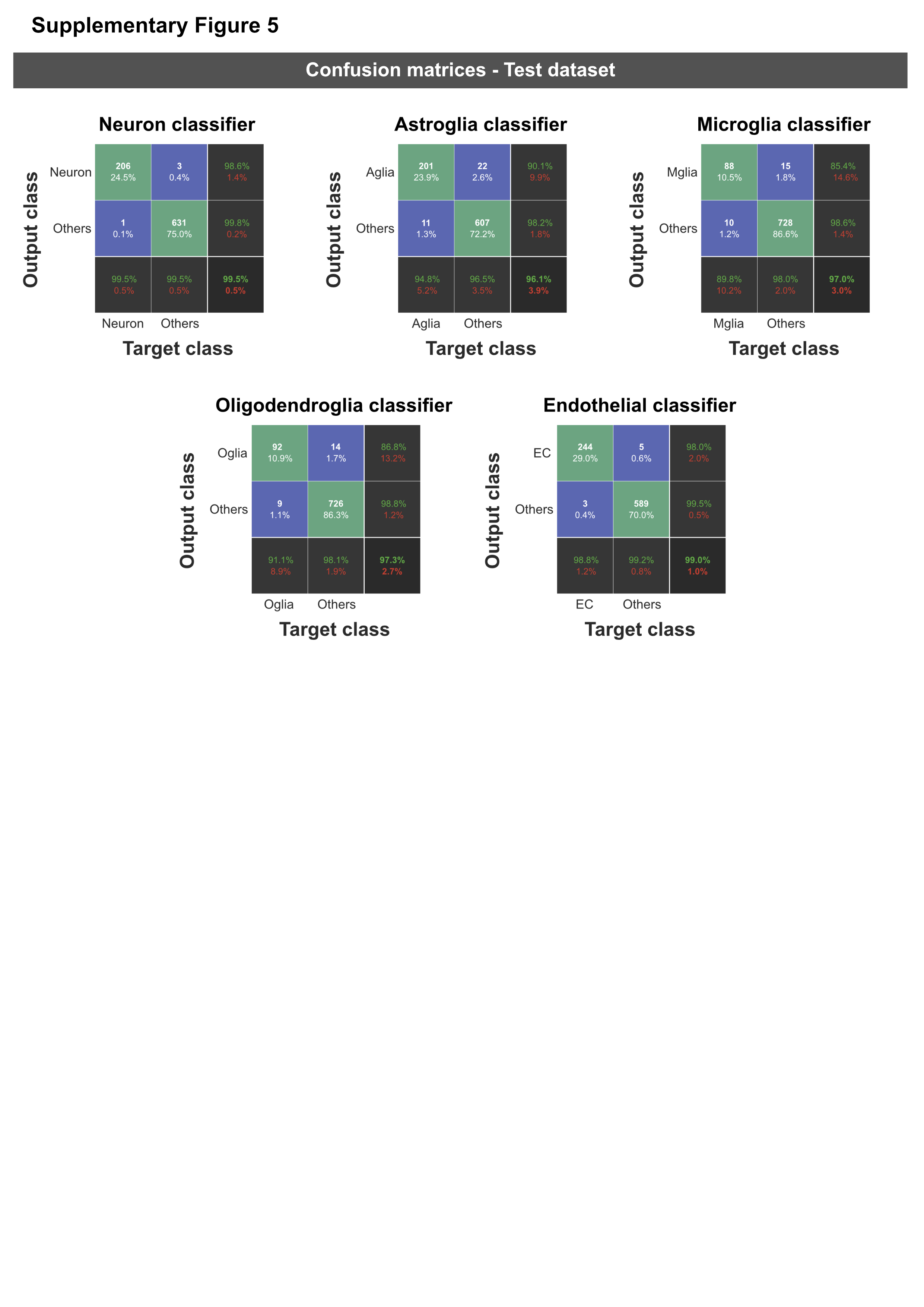

Supplement: S5 Fig — Since each classifier distinguishes between the desired class and every “other” class, the confusion matrix consists only of 4 fields. Rows show the predicted class (Output Class), and the columns show the true class (Target Class). Green fields illustrate correct identification of target and “other” class, blue fields illustrate erroneous identifications. The number of observations and the percentage of observations compared to the total number of observations are shown in each cell. Column on the far right shows the precision (or positive predictive value) and false discovery rate in green and red, respectively. Bottom row denotes recall (or true positive rate) and false negative rate in green and red. Cell on the bottom right shows overall accuracy of the classifier. Plot data can be found in S1 Data. (TIFF) [file pbio.3002357.s005.tiff]

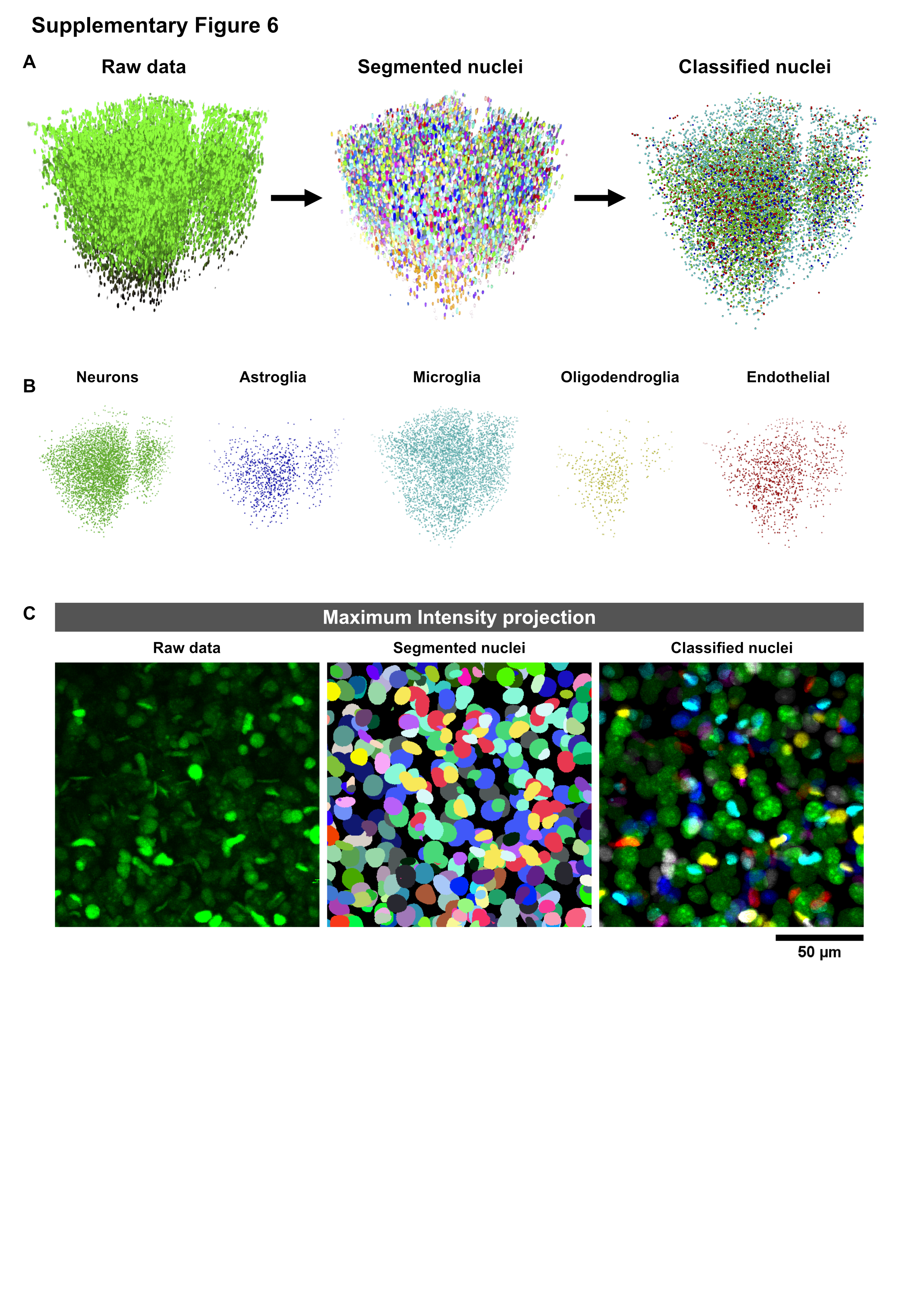

Supplement: S6 Fig — (A) Three-dimensional representation of raw, segmented, and classified data (700 μm × 700 μm × 700 μm volume) visualizing comprehensive cell type composition. (B) Cell type distribution in a single volumetric stack. (C) Maximum intensity z-projection of a sub volume (150 μm × 150 μm × 100 μm) showing raw, segmented, and classified nuclei in the X-Y axis. (TIFF) [file pbio.3002357.s006.tiff]

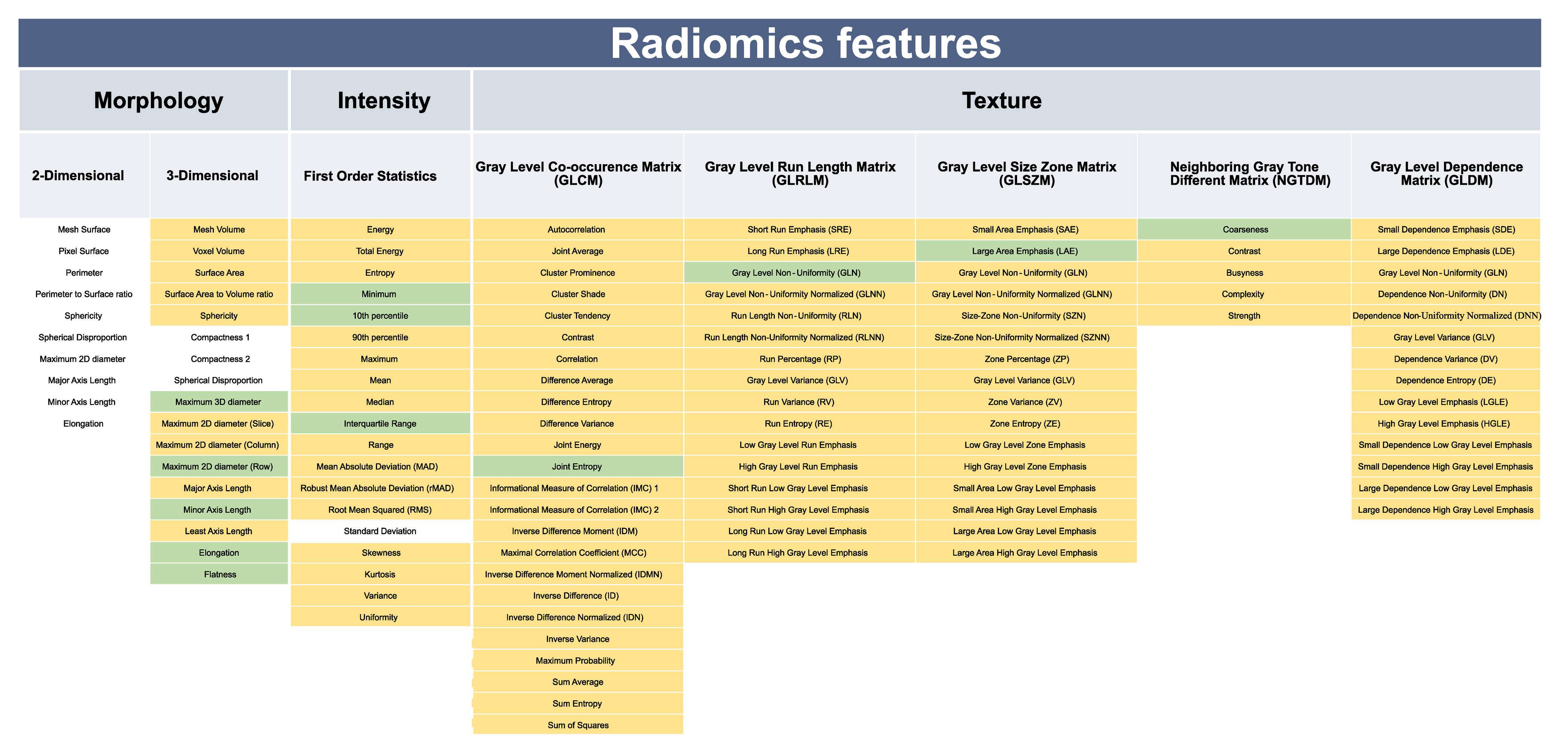

Supplement: S1 Table — Features marked in green were used for cell type training and classification. (TIF) [file pbio.3002357.s007.tif]

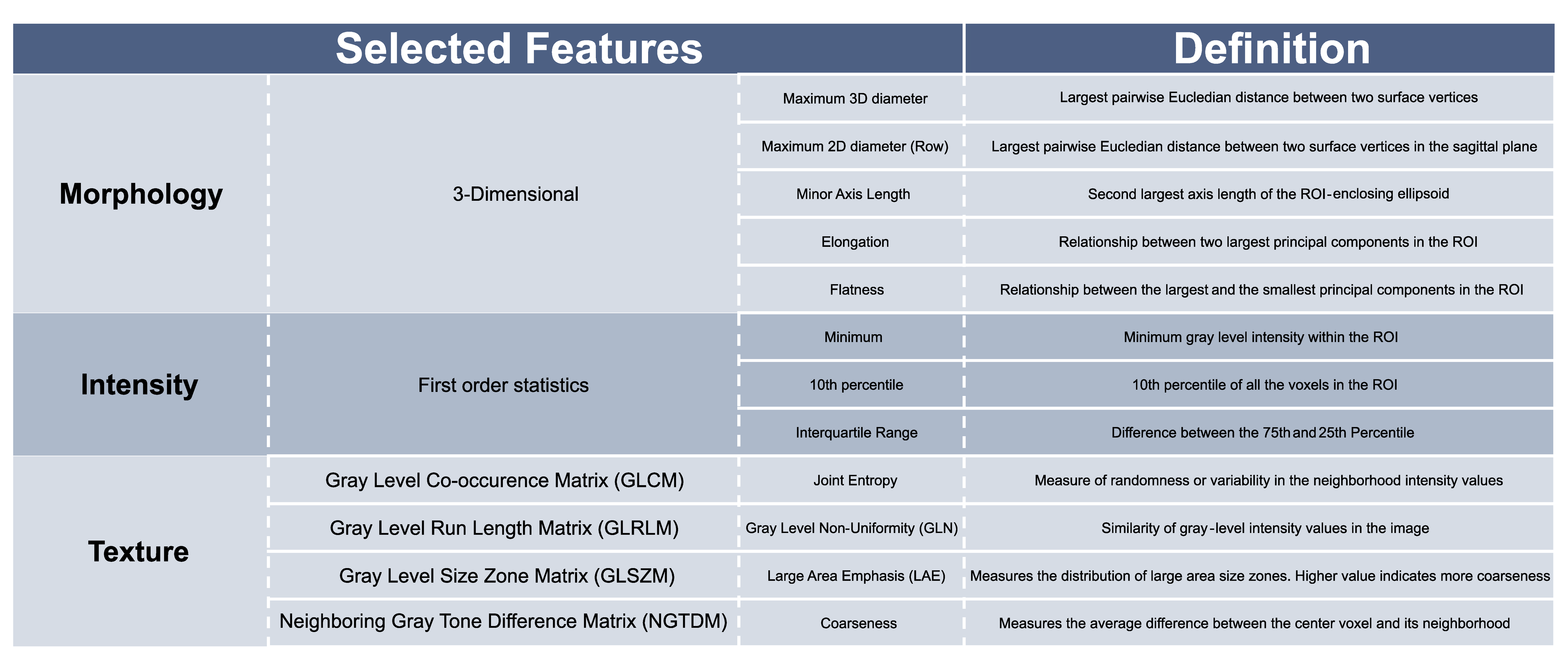

Supplement: S2 Table — (TIF) [file pbio.3002357.s008.tif]
