# Supplementary material for: Accurate classification of major brain cell types using in vivo imaging and neural network processing
Source: PLoS Biol. 2023 Nov 9;21(11):e3002357. doi: 10.1371/journal.pbio.3002357 (PMC10689024; doi:10.1371/journal.pbio.3002357)
Supplement: S1 Text — (DOCX) [file pbio.3002357.s009.docx]

Explanations for technical terms used in the manuscript:

**Accuracy:** Accuracy is a performance metric that quantifies the proportion of correctly classified data (both true positives and true negatives) out of all the data.

**Augmentation:** Augmentation involves applying various transformations, such as rotation, flipping, scaling, or adding noise, to the training data to increase its diversity. It helps improve the generalization and robustness of machine learning models.

**Binary** **mask**: A binary mask is a binary image where pixels are classified as either "foreground" (usually denoted as white) or "background" (usually denoted as black). Binary masks are commonly used to represent segmented regions, where each pixel is either part of the segmented structure (foreground) or not (background).

**Coarseness:** Coarseness is a texture feature that describes the scale of intensity variations within an image. It quantifies the contrast between regions of different intensities and their spatial frequency. A higher value indicates a lower spatial change rate and a locally more uniform texture.

**Confusion plots:** Confusion plots, also known as confusion matrices, are used to visualize the performance of a classification algorithm. They display the number of true positive, true negative, false positive, and false negative predictions made by the algorithm.

**Crops:** Crops refer to smaller regions or patches extracted from larger images.

**Entropy**: Entropy is a measure of the randomness or uncertainty in an image. It can be used to characterize the complexity of structures or textures within the image.

**Epoch:** An epoch is one complete cycle through the entire training dataset during the training process of a machine learning model in bioimage analysis.

**False discovery rate:** The false discovery rate (FDR) is a measure used to control the rate of false positive findings. It represents the proportion of incorrect positive predictions (false positives) among all positive predictions made by an algorithm.

**False positive / false negative:** False positive indicates an incorrect positive prediction, such as when the algorithm wrongly identifies background regions as part of the object of interest. False negative, on the other hand, refers to an incorrect negative prediction, where the algorithm fails to identify actual regions of interest.

**First order statistics:** First order statistics refers to the basic statistical measures computed directly from the pixel intensity values in an image, such as mean, median, standard deviation, etc.

**Flatness:** Flatness shows the relationship between the largest and smallest principal components in the region of interest (ROI) shape. The principal component analysis is performed using the physical coordinates of the voxel centers defining the ROI.

**Gray level co-occurrence matrix:** The gray level co-occurrence Matrix (GLCM) is a texture analysis method that quantifies the frequency of intensity value pairs at various spatial relationships within an image. It is commonly used to extract texture features.

**Gray level dependence matrix:** The gray level dependence matrix (GLDM) is a texture analysis technique that captures dependencies between pairs of pixels with specific intensity values in an image.

**Gray level run length matrix:** The gray level run length matrix (GLRLM) is a texture analysis technique that characterizes the lengths and frequencies of consecutive pixel runs with the same intensity value in an image.

**Gray level size zone matrix:** The gray level size zone matrix (GLSZM) is a texture analysis method that provides information about the distribution of connected regions of similar intensity values in an image.

**Gray level non-uniformity:** Gray level non-uniformity (GLNU) is a texture feature that quantifies the variance in pixel intensities within an image. It provides information about the spatial variations of grey levels in the image.

**Ground truth**: Ground truth refers to the manual or expert annotations of the correct regions of interest in an image. It serves as the gold standard for evaluating the performance of algorithms and machine learning models.

**Neighboring gray tone difference Matrix**: The neighboring gray tone difference matrix (NGTDM) is a texture analysis method that quantifies differences in pixel intensity values between neighboring pixels in an image.

**Overfitting:** Overfitting is a common problem in machine learning in general, where a model becomes too specialized on the training data and fails to generalize well to new, unseen data. It occurs when a model learns noise or irrelevant patterns from the training set.

**Precision:** Precision is a measure of the accuracy of positive predictions made by an algorithm. It calculates the proportion of true positive instances out of all positive predictions (true positives plus false positives).

**Radiomics:** Radiomics is a field in bioimage analysis that focuses on the extraction and analysis of quantitative features from images like texture, shape and morphology.

**Recall:** Recall, also known as sensitivity or true positive rate, is a measure of the algorithm's ability to correctly identify positive instances (true positives) out of all actual positive instances in the image.

**Segmentation:** Segmentation refers to the process of identifying and delineating regions of interest (ROI) within an image, separating them from the background or other structures. The goal is to partition the image into meaningful regions that correspond to specific biological structures or objects of interest.

**Training:** Training involves using a dataset with known ground truth annotations to teach a machine learning model or algorithm how to perform the task, such as segmentation or classification, on new, unseen images.

**True positive / true negative:** True positive refers to correctly identified positive instances (e.g., correctly identified regions of interest). True negative, on the other hand, refers to correctly identified negative instances (e.g., background regions correctly identified as not being part of the object of interest).

**Validation:** Validation is the process of assessing the performance and generalization capabilities of a trained algorithm on a separate dataset. It helps to ensure that the model's performance is consistent and reliable on new, unseen data.
