# Supplementary material for: Accurate classification of major brain cell types using in vivo imaging and neural network processing
Source: PLoS Biol. 2023 Nov 9;21(11):e3002357. doi: 10.1371/journal.pbio.3002357 (PMC10689024; doi:10.1371/journal.pbio.3002357)
Supplement: Attachment — Submitted filename: Response_To_Reviewers_Knabbe.docx [file pbio.3002357.s014.docx]

Reviewer #1: In this paper, Das Gupta et al describe an interesting approach for labelling major cell types in the brain using a computational method.

I think the approach is clever, and could potentially be useful for many researchers. However, this is a computational method paper without code, which I can never recommend for publication. The authors must share their code as an easily accessible package, including clear instructions, scripts with comments, example applications etc. I would ask that they share the code with the reviewers for peer review purposes, and then commit to releasing this code publicly at journal publication.

Similarly, the data should be made available publicly at publication date (it currently is not). The way the code is described in the methods makes me think there will be some work needed to put all the scripts and methods together into an easily accessible code package, and this is the work that I as a reviewer would like to request. I will make other comments below, which are of a secondary nature and much less important.

Dear reviewer #1, thank you very much for the positive feedback regarding our paper. During the revision process, we have made all code with examples publicly available, as well as all the data used for the publication. Please find code and examples here (https://github.com/adgpta/NuCLear), the data used for training of the classifiers as well as the raw data for the results can be found here: <https://heidata.uni-heidelberg.de/privateurl.xhtml?token=4c5be3dd-802a-4e9f-a7a6-931771380c96>

Other comments:

I think it should be stated very explicitly that this method can only categorize shallow cell types in the brain, i.e. neurons, astroglia, microglia etc. For the neuroscience community, cell types in the brain usually refers to types of neurons, such as "chandelier cells", or "PV-positive" cells, and these cannot be classified using this approach.

Thank you for the suggestion. As requested by the assigned editor, we expanded our method during the revision to include the possibility to distinguish between excitatory and inhibitory nerve cells and added supplementary figure 2 describing the results. We are convinced that it will be possible to add more specific cell types given there are enough ground truth data for training available.

Another thing that should be stated explicitly is that the brain is the only body region where a clear coverslip can even be implanted for in vivo imaging. This is very difficult to do for any other body organ, and the data quality from those implants is much much worse, precluding the application of these computational methods which need fine visual features to work.

We appreciate this suggestion. We still think that NuClear might be applicable in other organs as well, as in recent years technological advances have made it possible to perform in vivo imaging through chronically implanted coverslips in other rodent organs such as skin, spinal cord, abdominal organs, tongue and even lungs (see for an overview (Choi, Kwok et al. 2015), for a description of the procedure for acute in vivo lung imaging(Ueki, Wang et al. 2020)). We added these references along with a sentence: that good quality imaging data is needed for the method to work.

Another caveat of the method that should be stated is that it will depend on the type of microscope, depth of imaging and overall quality of the preparation. This makes a classifier trained on one set of data less robust at classifying new types of data, and it could explain the conflicting results which the authors report in the discussion with respect to the motor cortex volume imaged. To improve the ability of the decoder to generalize, the authors or other users could add augmentations to the training data to make it more similar to their own data, such as 3D blurring with PSF-shaped convolutions. This doesn't need to be done here, but I think it is important to give potential users options and ideas for what to do if the method does not work out of the box.

We added these sentences to the discussion: “For now, segmentation and classification depends on the type of microscope used, quality of the images, depth of imaging as well the region which was analyzed. To make the classification more robust and accountable for different qualities of fluorescence signal, augmentations to the training data could be added such as 3D blurring with PSF-shaped convolutions.”

Reviewer #2: The authors have presented a novel method for automatic classification of 5 major cell types of cortical layer 2/3: neurons, astroglia, microglia, oligodendroglia and endothelial cells. The method requires two neural networks, one for nuclei segmentation, the other for cell-type classification based on features of the nuclei. Both neural networks require training via ground-truth datasets, where the ground-truth datasets are created manually. After the initial training, the neural networks can be used in longitudinal studies where a given block of tissue is imaged over an extended period time. Hence, the NuCLear method presented by the authors could prove useful for researchers studying tissue composition in the brain and perhaps other types of tissue.

However, it is not clear what resources the authors intend to provide besides the information contained in the manuscript; NuCLear is not a software package but a two-step neural-network method for classifying major cell types.

We thank reviewer #2 for the extensive comments, which we have all addressed in the revised manuscript. The manuscript improved substantially due to the specificity of the comments.

Major Comments:

1. The abstract is short and heavily weighted to "selling" the authors' NuCLear method with overreaching claims and very little mention of their experimental results (one brief sentence, line 22-23). The authors have not included the type of animal used in the study, what part of the brain that was investigated nor what major cell types were classified.

We changed the abstract to incorporate brain regions that were imaged and the possibility to longitudinally image the same cells, thus dynamically monitoring changes. We also added a sentence mentioning experimental results beside the demonstration of the method.

2. The order in which the authors present their Results is confusing. This is not helped by everything being presented in two long paragraphs. It appears the authors have started their Results with an overall summary with 3 main points: volumetric imaging of nuclei (GFP), neuronal network training using GFP nuclei and ground-truth data created via tdTomato and mCherry, classification of 5 main cell types. Perhaps the authors can make it clear they are summarizing their method from beginning to end here, and the remaining Results will be about filling in the details. However, the authors have not included their method of nuclei segmentation in their initial summary. Finally, the section on "Automated segmentation of nuclei in the raw data" (line 91) comes after the section describing the generation of ground-truth datasets via tdTomato and mCherry (line 73). This may be confusing since generation of the ground-truth datasets requires segmentation of the nuclei. I would suggest the authors present their Results in the same order as their Methods.

Thank you for your addressing the organization of our Results section. We understand the confusion our initial structure might have caused. To address your concerns, we have made several adjustments to provide more clarity and coherence in our presentation. Firstly, we have made it explicit at the outset that the initial summary of the Results section encapsulates the overarching methodology we used from beginning to end. Finally, we have revised the order of our Results section to align more logically with the Methods section. This reordering will hopefully eliminate any confusion regarding the process sequence. We believe that these changes improve the flow and understanding of our Results section.

3. There is little information content in Figure 1. Moreover, the stripped-down simplicity of the cartoon seems to imply the NuCLear method is easy to use and requires few methodological steps. However, after looking at Figure 3a and Supplementary Figure 3, and reading the Methods, one begins to realise the numerous tasks necessary to perform the NuCLear method. There are actually two neural networks that are required, one for segmentation of the nuclei and the other for cell-type classification. Both neural networks require training via ground-truth datasets. Moreover, the ground-truth datasets have to be computed manually. I think Figure 1 should be replaced with Figure 3a or some version of it; it could also include information in Supplementary Figure 3. Or a simple flow box diagram could be useful.

Thank you for the suggestion, we changed Figure 1 to now include flow-diagrams to make the process of segmentation, classification and training easier to understand.

4. The authors' figures are inadequately labelled making it frustratingly difficult to read/review their manuscript. The panel labels in the figures (a, b, c…) often do not match the labels in their legends (the authors have apparently rearranged their figures without rearranging the legends). Several graphs are missing units. There are many panels that are not adequately described in the legends.

We are very sorry for the mistakes that happened during the preparation of the manuscript. They have been corrected in the revised version.

5. The authors may not have computed their densities correctly. On line 317 they state: "Nuclei touching the borders of the imaging volume were excluded." To compute density correctly they need to consider the border effect (Gundersen 1977). For a volume, this means 3 walls of their volume should be "exclusive" and the other 3 "inclusive" when counting the nuclei. Gundersen HJG. Notes on the estimation of the numerical density of arbitrary profiles: the edge effect. J Microsc. 1977 Nov;111(2): 219-223. doi: 10.1111/j.1365-2818.1977.tb00062.x

Thank you very much for this suggestion. The reason we excluded all nuclei touching the borders was to avoid mistakes in the classification due to nuclei being not fully inside the imaging volume. As the total volume of one imaging stack is around 700 µm³, containing around 20000 cells and we have around 600 border touching cells per volume (0.03 %), we would argue that the possible edge effect mistake is relatively low compared to the total number of cells in the whole volume.

6. From what I can tell from the Results the authors have only applied their method to one 3D volume of the cortex (line 129-130: "The 3D classification results for one example volume containing 20123 cells are shown in Figure 3E"). I would expect that after the authors trained their neural networks they would then move on to classifying neurons/glia in tissue that was not used in the training process, in multiple volumes as a proof of principle.

Thank you for this helpful comment. Indeed, we applied our method to in 16 volumes of 8 mice over two imaging time periods 12 weeks apart. We added a sentence to the results clarifying this more.

7. The authors have not discussed/explained why they did not attempt to classify neurons into subtypes as they did with glia. I would think the NuCLear method would find wider application if it was possible to categorize neuronal subtypes.

We added a possibility to subdivide the “Neuron” class into excitatory and non-excitatory cells during the revision (supplementary figure 2). We used CaMKII-mcherry and mDLX_NLS_mruby2 as markers for excitatory and inhibitory neurons, respectively. In principle and with the right amount of good quality training data, it may be possible to distinguish neuronal cells also into more specific subtypes.

8. The authors have not clarified how much training of the neural networks will be necessary to perform their NuCLear classification method. Will the training only be necessary at the beginning of a study and then be valid thereafter for weeks/months/years on multiple preparations? Could the neural networks be shared between labs? Or will it be necessary for each lab to train their own neural networks?

Thank you very much for this suggestion, we added the following sentence to the discussion: “Once a segmentation and classification network has been trained, it can be used for the full course of a longitudinal experiment and in different experimental settings if the conditions do not change the nuclear features. Sharing of classifiers between laboratories using similar imaging approaches will be possible as well.”

9. There is a lack of experimental quantification in methods, e.g. the number, age and type (male/female) of mice, the number of imaged volumes used in the various steps of the NuCLear method.

Thank you for this suggestion, we added the abovementioned information to the method section of our manuscript. In supplementary figure 4G we specified the number of reporter mice the training of each classifier.

10. It is not clear what data/code/networks (i.e. Resources) the authors plan to make available for others to use. Will a lab wanting to perform the NuCLear method have to replicate the authors' study from scratch?

We published the segmentation model, the classification model as well as a detailed guide with exemplary data online so that in principle everybody could either use our pretrained models for their own experiments or generate their own models. All data can be found here (https://heidata.uni-heidelberg.de/privateurl.xhtml?token=4c5be3dd-802a-4e9f-a7a6-931771380c96), all scripts and explanations can be found on our github page mentioned in the revised manuscript (https://github.com/adgpta/NuCLear).

11. There are several terms that are never defined in the manuscript: shape segmentation, binary mask, flatness, grey level non-uniformity, entropy, first order minimum, coarseness, overfitting, pixel entropy, confusion plots, radiomics, crops, etc.

Thank you for the suggestion. We added an appendix to the manuscript, explaining commonly used terms in the fields of image analysis and neural networks.

Specific (minor) Comments

Title:

I think "monitoring" is not the best word for the title. The paper is more about classification of cell types than about monitoring cells over time.

We appreciate this comment, however, we would still like to keep this title as we think that the possibility to monitor the cell type composition over a long period of time is one advantage over other published methods such as spatial transcriptomics.

Abstract:

Line 18. "Comprehensive analysis of tissue composition has so far been limited to ex-vivo approaches." This sentence is very general in scope and is not correct. I can think of MRI studies as one example.

We changed this sentence to focus more on the detailed analysis which is surely not possible with MRI.

Line 21. "This allowed us to classify all cells per imaging volume". This statement is not correct. There was a large number of unclassified and undecided cells in this study (Figure 3). And segmentation was not perfect.

Thank you for the suggestion. We changed the sentence in the abstract to: “Using NuCLear, we were able to classify almost all cells per imaging volume in the secondary motor cortex of the mouse brain (0.25 mm3 containing ~25000 cells) and to identify their position in 3D space in a non-invasive manner using only a single label throughout multiple imaging sessions.”

Line 23. "…using only a single label." This statement is not correct. Besides labelling the nuclei with GFP, the authors labelled individual cell types with tdTomato and mCherry.

Thank you for this comment. TdTomato or mCherry were used to generate ground truths for training the classifiers, during later experiments only mice with Histone-GFP were used.

Line 24. "NuCLear opens a window to study changes in relative abundance and location of all major brain cell types…" The phrase "all major brain cell types" may be misleading. On first reading I was anticipating the authors meant that their method would be used to classify major neuronal types (pyramidal, stellate, etc.) but in fact neurons are all grouped into one category.

Thank you for this suggestion. We changed this misleading phrase to: “. NuCLear opens a window to study changes in relative abundance and location of different cell types in the brains of individual mice over extended time periods, enabling comprehensive studies of changes in cellular composition in physiological and pathophysiological conditions.”

Line 25. "…enabling comprehensive studies of changes in cellular composition in physiological and pathophysiological conditions". If the pathology changes the shape of the nuclei then perhaps the authors' method will no longer work. The phrase "cellular composition" usually refers to intracellular composition, not cell-type composition of a tissue.

Thank you very much. We changed this sentence to: “NuCLear opens a window to study changes in relative abundance and location of different cell types in the brains of individual mice over extended time periods, enabling comprehensive studies of changes in cellular composition in physiological and pathophysiological conditions. “To avoid any misunderstanding, we added a paragraph to the discussion stating that for correct classification the shape of the nuclei needs to be similar in all imaging timepoints: “Pathophysiological conditions that alter nuclear shape and texture might influence the classification accuracy. “

Line 26. "NuCLear will work with any fluorescence-based microscopy and perform in any organ or model system." The authors should be more careful with their choice of words such as "all" and "any" and "every". Other fluorescence-based microscopy methods might not have the necessary resolution to allow segmentation and deep learning in 3D. Not all organs, or parts of the brain, can be accessed using a chronic window implant.

We removed the last sentence of the abstract.

Introduction:

Line 35: "all brain cell types" would be better as "multiple cell types".

We have changed “all brain cell types” to multiple cell types”.

Line 35-38: "…each cell type would require an individual labelling strategy using a set of specific promoters and fluorophores. This would require prioritizing cell types from the outset and thereby limit the scope to a small subset of the population". Are the authors implying their method resolves this problem? If that is so, then I am not sure how since their method has a labelling strategy using promoters and fluorophores.

Thank you very much for your comment. We probably did not emphasize enough, that our method only uses fluorophores and promotors for the training of the classifiers for each cell type. These classifiers are then used only in Histone-GFP mice without any other fluorophores. We rephrased some sentences to make this better understandable for the reader.

Line 46: "all brain cell types". See comment above (line 35).

We changed that sentence to “multiple cell types”.

Line 49-50: "in the same mouse" is probably not what the authors are implying here.

We mean “in the same mouse” as we are only using Histone-GFP mice to identify the cell types after training of the classifier beforehand.

Line 50: "…using only one genetically modified mouse line" should be "using a single genetically modified mouse line".

We have changed “only one genetically” to “using a single genetically”.

Line 51: "Histon" should be "histone". This typo occurs elsewhere.

Thank you very much for your comment, we changed all occurrences of “Histon” to “histone”.

Line 54-55: "In addition, knowing the 3D location of every cell enables previously inaccessible analyses." This sentence is vague and should be reworded to be more specific.

This has been reworded to: Additionally, determining the precise coordinates of every nucleus within the imaged space allows one to assess spatial relations of cells, e.g. the degree of cell clustering vs. even distribution of cells. This can be utilized as an indirect marker of glial territory size, which has been proven to be relevant in pathogenesis of disease such as Alzheimer's (Bouvier, Jones et al. 2016), or give information about neuron-glia-vasculature proximity (Zisis, Keller et al. 2021).

Line 56-57: "…the concept of this technique will be applicable to many other cellular imaging techniques and in different experimental systems…" This phrase is vague and should be reworded to be more specific.

This sentence has been rephrased to: “Apart from the application in longitudinal in vivo imaging of the mouse brain, the concept of this technique could be applicable to many other cellular imaging techniques such as confocal and widefield imaging of different organs, when ground truth data is available. This will make NuCLear a powerful approach for future analyses of large-scale automated tissue composition.”

Results:

Line 67: "…where known cell types were additionally labelled with a red fluorescent protein (tdTomato or mCherry)". It is not clear what "additionally labelled" means. The green label should be mentioned here.

We have rewritten this part of the results extensively. This sentence was rephrased to: “In addition to the histone 2B-eGFP-labeled nuclei, one red fluorescent marker protein was introduced (tdTomato, DyLight 549 or mCherry) to mark a specific cell type. The colocalization of both the red and green label allowed us to assign nuclei to the specific cell type and to extract ground truth data for nucleus classification in the green channel. "

Line 70: "The trained neuronal network was used to classify cell types based on their GFP-labeled nuclei." This sentence seems redundant with the previous sentence.

This sentence has been removed from the manuscript.

Line 71-73: "In our study we selected five different cell types for classification Neurons (yellow), astroglia (blue), microglia (green), oligodendroglia (turquoise), and endothelial cells (red)". A colon is missing after "classification". Colours would be better listed in the figure legend.

The colon has been added, color information has been added to the figure legend.

Line 73-76: "To generate ground-truth datasets without a bleed through of the strong tdTomato fluorescence towards the GFP-channel (Supplementary Figure 1F), we chose a method of inducing the expression of tdTomato for unequivocal cell type identification after imaging of the nuclei." I suggest starting a new paragraph here. Why is bleed-through a problem for tdTomata and not mCheery? The word "unequivocal" is not correct since type identification is not perfect.

We added a new paragraph here and also added information, that the mCherry fluorescence is more right shifted compared to the tdTomato fluorescence:

“To avoid a bleed through effect of the red fluorescent protein tdTomato into the eGFP channel, which could influence the quality of the classification, we induced the Cre-ERT2-dependent expression of tdTomato via intraperitoneal injection of tamoxifen (Supplementary Figure 1D, right) for cell type identification after the first imaging of the nuclei (Figure 2B, overlapping emission spectra for eGFP and tdTomato are shown in Supplementary Figure 1E, bleed through of the tdTomato signal into the eGFP channel is demonstrated in Supplementary Figure 1F)”

Line 76-78: "We selected a labelling strategy consisting of a tamoxifen-inducible cell type-specific Cre-recombinase in conjunction with…" The information in this sentence seems better placed before the previous sentence. A new paragraph could start: "To generate ground-truth datasets, we selected a labelling strategy consisting of…"

We changed the order of the sentences to incorporate this and the next suggestion as well.

Line 79-80: "We acquired volumetric images of nuclei from these mouse lines and induced tdTomato expression via intraperitoneal injection of tamoxifen." The authors could move their tdTomata bleed-through information to this sentence.

See above comment.

Line 81: "the same location" is singular, but in previous sentence volumetric imaging is plural.

Thank you very much for pointing this out. We have rephrased the entire results section to make it better understandable.

Line 81-83: "the previously imaged nuclei were selected by overlay with the red fluorescence signal." Readers may not understand this phrase. The authors should mention the green signal and those nuclei with both green and red overlap are selected. Was selection manual or automated?

We clarified this sentence and added the information that the nuclei were manually selected: “For the preparation of the ground truth datasets for the tdTomato expressing mice, nuclei were manually selected from the pre-induction timepoint, wherever the green and red fluorescence signals overlapped in the post-induction timepoint images (Figure 2C). For the microglial and neuronal datasets, nuclei that overlapped in the green and red channel were manually selected (Figure 2C, Supplementary Figure 1G, 6B).”

Line 83-84: "As microglia changed their positions after induction with tamoxifen…" Was this effect only for microglia? If so, the authors should clarify this.

To make it clearer, we changed the sentence to “As microglia were the only cells to change their positions after …”.

Line 88-90: "Hence, this approach was used to ascertain images of the labeled nuclei to be undisturbed by signals possibly arising from the red fluorescence used for unequivocal identification of the cell type." This sentence is confusing. Is it a summary sentence for all cell types or just neurons? As before, the word "unequivocal" is incorrect.

Thank you very much for pointing this out. We have now introduced the possibility to distinguish between inhibitory neurons and excitatory neurons and removed this sentence from of the manuscript.

Line 91: "Automated segmentation of nuclei in the raw data…" This is the first mention of segmentation and it is not sufficiently described here. At what stage(s) does segmentation occur during the NuCLear method?

During the revision, we added a sentence before this sentence briefly explaining the classification part of the NuClear method, where segmentation of nuclei is the first step. We shortly introduced the concept of segmentation. We also added an appendix to explain image analysis related concepts.

Line 106: "Having demonstrated the ability of nuclear features to distinguish between their corresponding cell types…" The authors should begin a new paragraph here.

We added a new paragraph.

Line 108-100: "Using data from the five reporter mouse lines, we trained one classifier per cell type for distinguishing one cell type from the respective other cell types." This sentence is difficult to understand and should be reworded.

We rephrased this sentence to: “Utilizing the dataset obtained from the five reporter mouse models, a single neural network classifier was trained for each cell type. The purpose of each classifier was to distinctly differentiate its corresponding cell type from the diverse array of other cell types. “

Line 110-111: "To increase the amount of training data and equalize the nucleus counts for each cell type to reduce training bias…" This phrase has run-on "to" prepositions.

We rephrased the sentence to: “To increase the amount of training data and equalize the nuclei counts for each cell type, thus reducing training bias, synthetic data was generated from the features of the original dataset. “

Line 114-115: "After classifying the whole dataset, nuclei were either assigned to a single class, two or more classes (undecided) or to none (unclassified)." The phrase "After classifying the whole dataset" is confusing: this sentence is about classifying. The authors should remind the reader what their different classes are.

We rephrased this sentence to: “After each nucleus was classified by all trained classifiers, it was either assigned to a single class (neurons, astrocytes, microglia, oligodendroglia, endothelial cells), two or more classes (undecided) or to none (unclassified).”

Line 116-117: "Precision and recall rates for each classifier were high for neurons and endothelial cells." "Precision" and "recall" are not defined. Reference to Figure 3 in next sentence should be moved to this sentence.

We moved the reference to this sentence. Precision and recall are explained in the newly added appendix as well.

Line 117-118: "After combining all classifier output…" It is not clear what this means.

The sentence has been rephrased to “The classification accuracy for the entire training dataset was highest for neurons (98 %) and endothelial cells (99 %), whereas the classifier showed lower accuracies for all glial cell types, especially for microglia (96 %) (Figure 3C, D, for the respective confusion plots see Supplementary Figure 5). “

Line 118: "Accuracy" is not defined. Readers may not understand the difference between precision and accuracy.

We explained accuracy in the newly added appendix.

Line 129-130: "The 3D classification results for one example volume containing 20123 cells…" The authors should start a new paragraph here.

We added a new paragraph.

Line 130: I do not find Figure 3E and supplementary Figure 6 "intuitive".

We reworded this sentence to: “The 3D classification results for one example cortical volume containing 20123 nuclei (Supplementary Figure 6A,B) visualizes the relative abundance and positioning of cell types within the volume and their mutual spatial relationship (see also Supplementary Movie 1 for an animated version of the segmentation and classification process).”

Discussion:

Line 150: "all cells" is not correct.

We changed it to “cells”.

Line 153: "feasible" is not the correct word to use here.

We removed “feasible”.

Line 157: "perturbations, treatments, or adaptive processes." These could be clarified, especially "adaptive processes".

We have changed this sentence to “and in response to perturbations, treatments, or adaptive processes such as learning or exploring.”

Line 158-159: "…the best classification results regarding total classification accuracy and precision…" The second "classification" is redundant.

The second “classification” was removed.

Line 171: "expressed increasingly" should be reworded.

We reworded it to: “and has a higher expression. “

Line 173: "concluded" is not the correct word to use here.

This sentence was rephrased to: “Additional confirmation of the classifier's precision can be inferred by examining characteristics of the nuclei, like average nuclear volume and the surface-to-volume ratio, which were not utilized in the training phase.”

Line 177-190: "When applying the classification model to a Layer 2/3 volume…" This paragraph could be incorporated into the authors' Results.

Thank you very much for the comment. We still think that this paragraph is appropriately located in the discussion, as we compare our results with previously published data.

Line 178: "…extrapolating the number of cells per mm³…" It is not clear what this means.

We exchanged “extrapolate” with “calculate”.

Line 196-197: "When analyzing the effect of time on the imaging volumes from the secondary motor cortex…" I could not find the "time" aspect in the Results or figures.

In Figure 3 H, I, J we analyzed cell type numbers of the same locations in 8 different mice 12 weeks apart, studying the effect of time on the count of cell types in these regions. To make this clearer, we rephrased this sentence to “When analyzing the same imaging volumes from the secondary motor cortex 12 weeks apart, we were able to detect a trend towards astrogliosis, which might be due to the aging process leading to a higher GFAP-reactivity. ”

Line 204-205: "Our study illustrates only one possible application of NuCLear and subsequent analysis of 3D issue composition." The authors have not presented an application of their method, only details of the method itself.

Thank you very much for this comment. We applied the NuClear method to the same imaging volumes in 8 mice 12 weeks apart and analyzed changes in the cell type composition of this imaging volume. We are convinced that this counts as a simple application of this method, more complicated applications would include for example the application of toxic substances and analysis of the consequences for each cell type over time.

Line 211: The word "easily" is not correct.

We removed the word “easily”.

Line 214-215: "We anticipate that any cell type will be detected…" This is a bold claim.

To make the claim less bold we rephrased this sentence to “We assume that it will be possible to detect more cell types by adding classifiers trained with appropriate ground truth data.”

Line 216: "…we propose an easy and readily usable method…" It does not seem to me the authors' method is easy nor readily accessible. It requires someone trained in neural networks.

During the revision process we added detailed step-by-step instructions on our github page together with exemplary data to help implementing our method. We are convinced that this will make the method easily usable.

Line 217: The authors have mentioned the use of their method for "different organs" in multiple places but have not clarified what organs they are referring to. Most organs are not accessible for in vivo imaging.

We added a sentence to the discussion, citing a review from 2015 where organs accessible for chronic in vivo imaging are listed: “NuCLear will be applicable to different organs as techniques have emerged in the last years to perform in vivo imaging through chronic windows in other rodent organs such as skin, abdominal organs, the tongue or spinal cord (Choi, Kwok et al. 2015), depending on the image quality and the possibility to acquire images during multiple timepoints”.

Materials and Methods:

Line 275: "Two photon imaging was carried out with a two-photon microscope". "Two photon" is redundant.

The first “two photon” was removed.

Line 303-304: "The StarDist model was trained to segment nuclei in 3D using an in-house developed Jupyter Notebook". There does not seem to be enough information here for other labs to develop their own in-house segmentation model.

During the revision, we published a GUI-based version of the notebook that was used for training of the segmentation. It can be found on this github page: https://github.com/adgpta/NucleusAI/. We added this information to the manuscript. Using this software, readers should be able to replicate the steps leading to a segmentation model. Additionally, StarDist 3D <https://github.com/stardist/stardist>) was developed by the time the manuscript was finished. Methods are explained in the authors GitHub page.

Line 304: "Manually segmented and labelled nuclei…" It was not explained how nuclei were manually segmented/labelled. This may be non-trivial.

We added a sentence describing the steps for manual segmentation and labelling of the nuclei: “Nuclei were manually traced and segmented using the segmentation editor in Fiji (Schindelin, Arganda-Carreras et al. 2012).”

Line 313: "Feature extraction using PyRadiomics". The authors should add "nuclei" to the header.

We changed the header to: “Feature extraction of segmented nuclei using PyRadiomics”

Line 315: "…for each segmented nucleus…" The authors do not explain which nuclei dataset they are using here.

Extraction of all features were performed during the classification workflow as well as for the training of the classifier models. We have rephrased the sentence to: “extracted for each nucleus after segmentation using StarDist, including, but not limited, to morphological features…”

Line 320: "For supervised training of the deep neural network for cell type classification…" This phrase has run-on "for" prepositions.

This sentence was rephrased to: “To perform supervised training of the deep neural network for cell type classification, a ground truth dataset was created using the two-photon volumetric fluorescence data, automatically segmented nuclei, which were assigned a unique label, and the radiomics features.”

Line 324: "label ID" is not defined.

The first sentence was changed to define the term “label”: “To perform supervised training of the deep neural network for cell type classification, a ground truth dataset was created using the two-photon volumetric fluorescence data, automatically segmented nuclei, which were assigned a unique label, and the radiomics features.”

Line 327-328: "To increase the number of nuclei for training and make it equal in size for all cell types to avoid a bias in training…" This phrase has run-on "to" prepositions.

The sentence was rephrased to: “To increase the number of nuclei for training and make it equal in size for all cell types and thus avoid a bias in training, synthetic datasets were created from nuclei features using the synthetic…”

Line 336: "manual validation was performed in ImageJ/Fiji". The method for this was not explained.

We rephrased this sentence to explain the method better to: Detection accuracy and precision were calculated after visualization of raw data and segmentation in ImageJ/Fiji. True positive nuclei (TP), false positive nuclei (FP) and false negative nuclei (FN) were manually counted. 4 arbitrary volumetric images were selected containing up to 80 nuclei per crop. Accuracy was calculated as TP / (TP + FP + FN). Precision was calculated as TP / (TP + FP).

Line 353: Do the authors have a reference for the "adam" adaptive moment estimation optimizer?

We added a citation to the original publication introducing the adaptive moment estimation optimizer.

Line 356: It is not clear what "epoch" refers to.

A training epoch in the context of a neural network refers to a complete pass or iteration through the entire training dataset during the training process. We added a sentence, describing what “epoch” means. We also explained the term in the newly added appendix.

Line 361: "all nuclei" is not correct.

“All” was removed.

Line 363: "every segmented nucleus" should be "the segmented nuclei".

We changed “every segmented nucleus” to “the segmented nuclei”.

Line 364: "Every segmented nucleus" should be "The segmented nuclei".

We changed “every segmented nucleus” to “the segmented nuclei”.

Line 360-367: This section is confusing as it mostly repeats the previous methods.

We agree, that this mostly repeats previous methods. Nonetheless, in this paragraph the actual classification process is described, the methods before were focused on the training of the classification. We think it is appropriate, to keep this part as a short description of the classification process.

Line 374: the authors have not specified what type of t-test they used.

Thank you very much for this comment. We rephrased this sentence to: “Since the distribution of the data was non-parametric, the Wilcoxon signed-rank test was used for all statistical testing and p-values were corrected for multiple comparisons using the “bonferroni”-method”

Figures:

Figure 1: "all cell nuclei" is not correct. Missing information: mouse, type of mouse, type of fluorescence. There is no "c" label in the figure. Cell types and colours are not defined. It looks like the endothelial cells line the blood vessels but this is not explained by the authors.

Thank you very much for the comment. We changed “all cell nuclei” to “cell nuclei”. The requested information was added as well. As endothelial cells are defined as cells of blood vessels, we do not think it is necessary to mention that in addition.

Figure 2: Labels in legend do not match those in the figure. There are no "3D renderings" in the figure. Diameters have no units. It is not clear what "algorithm" the authors are referring to in the legend. The results in this figure may be confusing since the nuclei have been separated into cell types (panels e and f) before the authors have explained how they classified their neurons via neural networks (Figure 3).

We are very sorry for these mistakes. We changed the figure to include a simple flow chart describing the training process. Wrong labels have been changed. We added the nucleus diameter unit to the legend of Figure 2: “3D diameter in voxels, (voxel resolution in xyz: 0.29 µm x 0.29 µm x 2µm)”.

Figure 3a: It is not clear what is plotted under "radiomics features" and "neural network classifier". There are no units for volumes or surface-to-volume ratios.

We have changed Figure 3A to visualize the training process in a better way and added the following text to the legend: “Schematic depiction of the training process. After segmenting the raw data with StarDist for every cell type, radiomics features of nuclei were extracted from the ground truth data (blue) and synthetic data (green) was generated. For each cell type, a single neuronal network was trained to distinguish the corresponding nucleus type from the other nuclei.” We also added units for the volumes and surface-to-volume ratios to the figure legend.

Supplementary Figure 3: "A sequential forward feature selection algorithm was applied." I am not sure if this algorithm was mentioned in the manuscript.

We have mentioned the algorithm in the methods section: “Initially, a sequential feature selection algorithm “sequentialfs” was applied to the whole dataset to extract 12 features with the highest variability to reduce data overfitting. “

References:

Choi, M., S. J. Kwok and S. H. Yun (2015). "In vivo fluorescence microscopy: lessons from observing cell behavior in their native environment." Physiology (Bethesda) **30**(1): 40-49.

Ueki, H., I. H. Wang, D. Zhao, M. Gunzer and Y. Kawaoka (2020). "Multicolor two-photon imaging of in vivo cellular pathophysiology upon influenza virus infection using the two-photon IMPRESS." Nat Protoc **15**(3): 1041-1065.
