# Supplementary material for: Accurate classification of major brain cell types using in vivo imaging and neural network processing
Source: PLoS Biol. 2023 Nov 9;21(11):e3002357. doi: 10.1371/journal.pbio.3002357 (PMC10689024; doi:10.1371/journal.pbio.3002357)
Supplement: Attachment — Submitted filename: Response_To_Reviewers_Knabbe.docx [file pbio.3002357.s015.docx]

Reviewer #1: The authors have addressed my concerns. I do not have any follow-up concerns.

Dear Reviewer #1,

I would like to sincerely thank you for taking the time to review our manuscript. I'm gratified to learn that you are satisfied with the revised version and appreciate your positive feedback.

Your insightful comments during the review process have been invaluable in enhancing the quality of our work. The fact that you have no further suggestions gives us confidence in the improvements we've made.

Reviewer #2 (Jason Seth Rothman, signs review): "Comprehensive monitoring of tissue composition using in vivo imaging of cell nuclei and deep learning". The latest version of the authors' manuscript has improved considerably from their initial version. The authors have addressed all of my major and minor concerns. I have only a few remaining minor concerns about the revised manuscript.

Dear Dr. Rothman,

I would like to express our gratitude for your thorough and detailed review of our manuscript. Your extensive feedback has played an instrumental role in refining our work. We understand and appreciate the time and effort it requires to delve into a manuscript with such depth. Each of your comments has provided us with valuable insights, prompting necessary refinements and clarifications that have significantly improved the quality and coherence of our paper. We have made every effort to address your suggestions.

Line 18. "Comprehensive analysis of tissue composition has so far been limited to ex-vivo approaches." The authors state they changed this sentence but it has not changed. My example of MRI was to highlight how vague this sentence is, which does not even include the word 'imaging'.

We are very sorry for this mistake. We changed this sentence in the revised version as follows to also capture the imaging aspect: “Comprehensive analysis of tissue cell-type composition using microscopic techniques has primarily been confined to ex-vivo approaches.”

Lines 26 and 147: 'relative abundance' would be better as 'relative density'.

Thank you very much for this suggestion, we changed it accordingly.

Line 28: 'cellular composition' would be better as 'tissue composition' or 'cell-type composition' as used elsewhere in the manuscript.

We changed cellular composition to cell-type composition.

Line 32-34: 'Understanding the plasticity and interaction of different brain cell types in vivo has been a limiting factor for the investigation of large-scale structural brain alterations associated with diverse physiological and pathophysiological states.' This sentence is confusing. It is not clear what the 'limiting factor' is.

Thank you very much for this comment. We changed the sentence as follows to make it less confusing: “Understanding the plasticity and interaction of different brain cell types in vivo for the investigation of large-scale structural brain alterations associated with diverse physiological and pathophysiological states has been challenging due to the inability to study multiple cell types simultaneously”.

Line 39-40: 'studies assessing whole tissue composition quantitatively' would be better as

'quantitative studies assessing whole tissue composition'.

We have changed the sentence according to your suggestion.

Line 47: 'composition' would be better as 'tissue composition'.

We changed ‘composition’ to ‘tissue composition’.

Line 50: 'in the same mouse in vivo' would be better if the authors added 'over time', or some longitudinal aspect of their study.

We added ‘over time’ to the sentence.

Line 75-76: 'feature extraction' would be better as 'feature extraction of the segmented nuclei'.

We changed the sentence accordingly.

Line 97: 'right-shifted' might be better as 'red-shifted'.

We changed ‘right-shifted’ to ‘red-shifted’.

Line 101-103: 'nuclei were manually selected from the pre-induction timepoint, wherever the green and red fluorescence signals overlapped in the post-induction timepoint images.' This phrase could be changed to: 'nuclei were manually selected from the pre-induction timepoint images (green fluorescence signal) wherever they overlapped the post-induction timepoint images (red fluorescence signal).'

We have changed this sentence as follows: “To prepare the ground truth datasets of tdTomato expressing mice, nuclei were manually selected from the pre-induction timepoint images (green fluorescence signal) wherever they overlapped with tdTomato signal in the post-induction timepoint images (red fluorescence signal).”

Line 128-129: 'a single neural network classifier was trained for each cell type' would be better as 'five neural network classifiers were trained for each cell type'.

Thank you very much for this suggestion. We rephrased the sentence as follows: “Utilizing the dataset obtained from the five reporter mouse models, five neural network classifiers were trained, one for each cell type.”

Lines 152-158: 'When the trained model was applied, two features not chosen by the automated feature selection algorithm differed significantly between the classes…' These two sentences are confusing here since it is not clear how they relate to the 'longitudinal' topic of this paragraph.

To make it less confusing, we rephrased the sentence as follows: ‘To apply NuClear in mice, we studied 16 imaging volumes from 8 mice (male, 12-14 weeks at baseline) in layer 2/3 of the secondary motor cortex 12 weeks apart. When the trained model was applied to the first imaging timepoint (baseline), two features not chosen by the automated feature selection algorithm differed significantly between the classes: nucleus volume (Figure 3E) and mean surface to volume ratio, a measure for the sphericity of the nucleus (Figure 3F).’

Line 182: 'z-step size' would be better as 'axial resolution'.

We changed ‘z-step size’ to ‘axial resolution’

Line 183: 'more than 20000 cells' would be better as '~25000 cells' as stated earlier in the manuscript, or something similar.

We changed ‘more than 20000 cells’ to ‘~25000 cells’ as suggested.

Line 182-186: 'We were able to image a whole 3D volume of 700 μm³ consisting of more than 20000 cells in around 20 minutes, making it possible to perform large scale data acquisition in a repeated manner, enabling longitudinal intravital monitoring of tissue histology over time and in response to perturbations, treatments, or adaptive processes such as learning or exploring.' It is not clear whether the text following with 'making it possible to perform…' describes what the authors actually did in their study, or whether they are suggesting these methods are hypothetically possible. If it's the latter, then this sentence would be better if it was broken into two different sentences so there is no confusion.

As we indeed did not perform monitoring of tissue histology in response to perturbations, treatments or adaptive processes, we broke the long sentence into two different sentences to make it less confusing.

Line 368: 'Nuclei touching the borders of the imaging volume were excluded.' The authors state in their reply that the error introduced by their counting method is small. They could include their reasoning here in the Methods, which would be helpful to others who decide to use the authors' methods.

Thank you very much for this valuable suggestion. We added an explanation according to our response: “Nuclei touching the borders of the imaging volume were excluded to avoid mistakes in the classification. Within an imaging volume of 700 µm³ containing ~25000 nuclei, the number of border touching nuclei represented only 0.03 % of the total number of nuclei, thus diminishing the possibility of any error caused by an edge effect.”

Line 376: 'This allowed for identification of the label… ' It is not clear what label the authors are referring to here or why this label has not been identified. The previous mention of a 'unique label' indicates the labels were assigned manually and therefore should be known.

We rephrased this paragraph to make the meaning of it clearer: “With the help of the red fluorescence channel images, nuclei belonging to a specific cell type were manually identified. Nuclei that were overlapping in the green and red channel images in the post-induction timepoint were reidentified in the green channel images of the pre-induction timepoint. These nuclei were manually selected in the segmented StarDist output images. Using their label, corresponding radiomics features could be extracted from the pre-induction timepoint image (Supplementary Figure 4B).”

Line 406: "sequentialfs" should be "sequentials".

We refer to the Matlab function “sequentialfs” (<https://de.mathworks.com/help/stats/sequentialfs.html>). To make this clearer, we changed the sentence as follows: “Initially, a sequential feature selection function “sequentialfs” (MATLAB R2022b) was applied to the whole dataset to extract 12 features with the highest variability to reduce data overfitting”.

Figure 1B. There is a box for 'Trained network models' that is followed by what appears to be the trained network models. This looks redundant.

Thank you very much for this suggestion, we removed the box for ‘Trained network models’ in Figure 1, making it less redundant.
